# Novel approach to HER2 quantification using phosphor-integrated dots in human breast invasive cancer microarray

**DOI:** 10.1371/journal.pone.0303614

**Published:** 2024-05-15

**Authors:** Naoya Saito, Tsukasa Matsuo, Hitoshi Tsuda, Hiroyuki Yokota, Hisatake Okada

**Affiliations:** 1 Technology Development Headquarters, Advanced Core Technology Center, Konica Minolta, Inc., Hachioji, Japan; 2 Department of Basic Pathology, National Defense Medical College, Saitama, Japan; Qatar University College of Medicine, QATAR

## Abstract

HER2 expression in breast cancer is evaluated to select patients for anti-HER2 therapy. With the advent of newly approved HER2-targeted drugs for low HER2 expression breast cancer, more solid evidence on the whole spectrum of HER2 expression is needed. In this study, we quantitatively assessed HER2 expression from the whole core by combining high-intensity phosphor-integrated dot (PID) immunostaining and whole slide imaging (WSI) analysis. Two types of staining were performed using a 170-core tissue microarray of invasive breast cancer. First, HER2 was stained by immunohistochemistry (IHC), and IHC scores were determined by two practicing pathologists according to the ASCO/CAP HER2 guideline. Second, HER2 was stained with PID, and tentative PID scores were determined by quantitative analysis. The results show that PID can numerically classify HER2 expression status into scores 3+, 2+, 1+, and 0. The HER2 value quantified by PID strongly correlated with the 3, 3’-diaminobenzidine (DAB) IHC score determined by pathologists (R^2^ = 0.93). PID IHC score 1+ cases included both DAB IHC score 1+ and 0 cases, and low HER2 expression cases appeared to be often evaluated as DAB IHC score 0. Therefore, digital image analysis by PID and WSI can help stratify HER2 IHC. It may also help classify low HER2 expression.

## Introduction

Breast cancer (BC) is one of the most frequently diagnosed malignancies in women worldwide [1]. Human epidermal growth factor receptor 2 (HER2) is overexpressed or amplified in nearly 20% of BC cases and is an FDA-approved predictive marker to identify responders to anti-HER2 agents [2]. Immunohistochemistry (IHC) is currently the most commonly used assay to determine the HER2 status in BC [3]. HER2 expression assessment relies on pathologist scores according to ASCO/CAP guidelines [4]. Because of the semiquantitative nature of HER2 status assessment using HER2 IHC assays, HER2 IHC-equivocal cases might be HER2 negative and would not receive further HER2 status assessment [5]. The emergence of antibody-drug conjugates (ADCs) has shown efficacy in not only tumors with HER2 overexpression but also low HER2 tumors [6]. Conventional HER2 IHC assays have challenges, including not being calibrated to detect low HER2 levels and poor reproducibility of low HER2 expression diagnoses among pathologists [7, 8]. Therefore, HER2 expression quantification is becoming increasingly important.

Accurately predicting the efficacy of anti-HER2 therapies for BC may require methods to quantify HER2 receptor molecules. In recent years, several novel IHC methods to quantify HER2 levels are being investigated, including the quantum dot (QD)-based detection method [9], immunofluorescence method to quantify single-cell levels [10], IHC technique with QD-conjugated trastuzumab [11], quantitative IHC (qIHC) [12], HERmark Breast Cancer assay [13], and quantitative immunofluorescence AQUA method [14, 15]. In addition, quantitative digital image analysis using whole slide imaging (WSI) is investigated as a new method to assess HER2 IHC [16–20]. Whole-tumor section image analysis significantly improves between-pathologist reproducibility and is the optimal approach for clinical-based image analysis algorithms.

Muotafi et al. developed an assay that is complementary to conventional methods for the identification of low HER2 unamplified tumors through a combination of quantitative immunofluorescence and mass spectrometry [14]. However, they used Cy5 tyramide and anti-HER2/ErbB2 (clone 29D8) antibody for HER2 detection. Tyramide signal amplification (TSA) is one of the most commonly used approaches to increase the signal of IHC staining patterns; however, excess fluorochromes can precipitate into tissues and cause excessive background [21]. Primary antibodies are also different from antibodies approved for diagnostics.

The first feature of our study is the use of bright fluorescent particles. Our proprietary phosphor-integrated dots (PID) are organic fluorophore assembly-conjugated nanoparticles with features of high brightness, high photostability, and wide dynamic range (10^−6^ to 10^−1^ mM) [22]. PID has been used for HER2 detection in BC, Colony Stimulating Factor 1 Receptor (CSF1R) detection in tumor-associated macrophages [23], quantitative detection of Estrogen receptor alpha (ERα) [24], and intratumoral pharmacokinetics of new HER2-targeted ADC [25]. Furthermore, we are attempting to enhance the sensitivity of PID by devising applications [26, 27]. The second feature is the use of WSI. A previous study used 108 BC cases to calculate the PID score/ROI 100 μm^2^ using five randomly selected fields from each section [28]. Collecting the whole core invasive area using WSI is important to obtain accurate information because the previous method provides only partial information on five field of view.

The significance of this study is to stratify tissue microarray (TMA) with various HER2 expression levels by comprehensively visualizing and quantifying HER2 using PID and WSI.

## Materials and methods

### TMA

BC TMA (104 cases, duplicate cores) was purchased from US Biomax (Catalog# BR20810; Rockville, MD, USA). The TMA was used to define the distribution of HER2 expression in BC.

### IHC by DAB staining

IHC for PATHWAY anti-HER-2/neu (4B5) Rabbit Monoclonal Primary Antibody (ready to use, Catalog# 790–2991; Roche Diagnostics, Tucson, AZ, USA) was performed on the TMA sections by Ventana BenchMark GX using iView DAB universal kit (Catalog# 760–041; Roche Diagnostics). The iView DAB universal kit was used because it has no difference in sensitivity from the Ultra-View DAB universal kit and is routinely used by inspection companies. IHCs without primary antibodies were performed as negative controls.

### Evaluation of DAB

Scoring of the 4B5 IHC staining was performed independently by two experienced pathologists. HER2/neu expression was graded according to ASCO/CAP HER2 testing guidelines [3]: 0, no staining is observed or membrane staining is incomplete and is faint/barely perceptible in ≤10% of tumor cells; 1+, incomplete membrane staining that is faint/barely perceptible in >10% of tumor cells; 2+, weak-to-moderate complete membrane staining observed in >10% of tumor cells, or circumferential complete and intense membrane staining in ≤10% of tumor cells; and 3+, circumferential complete and intense membrane staining in >10% of tumor cells.

### PIDs

PID is prepared by perylene diimide assembly-conjugated nanoparticles [22]. The particle size of the fluorescent nanoparticles PID is approximately 130nm (excitation wavelength, 580 nm; emission wavelength, 620 nm). The surface of the nanoparticles was modified with streptavidin via polyethylene glycol (PEG).

### IHC by PID

TMA was deparaffinized and rehydrated, and then antigen retrieval was performed by boiling for 40 min at 95°C with BOND Epitope Retrieval Solution 2 (AR9640; Leica biosystems, Newcastle Upon Tyne, UK). The TMA was blocked using an in-house blocking solution (50 mM Tris-HCL, 3% BSA, 0.6% α-casein, 0.6% β-casein, 1% Tween-20 and 0.065% sodium azide) for 15 min at room temperature. After blocking, the TMA was incubated with a PATHWAY anti-HER2/neu (4B5) rabbit monoclonal primary antibody overnight at 4°C, followed by DISCOVERY universal secondary antibody (Catalog # 760–4205; Roche Diagnostics, 1:10) for 30 min at 25°C. Then, the TMA was treated with 0.27 nM PID (130 nm) for 2 h at 25°C. The secondary antibodies and PID were diluted with an in-house blocking solution. The TMA was fixed for 30 min in 4% PFA before being stained with hematoxylin (Sakura Finetek Japan, Tokyo, Japan) and mounted in malinol (Muto Pure Chemicals, Tokyo, Japan).

### Imaging by WSI

The WSI of HER2 (fluorescence field image) and hematoxylin stains (bright-field image) were captured by NanoZoomer S60 (Hamamatsu Phototonics, Hamamatsu City, Japan) at 20× magnification and saved in their image format (TIF). The focal plane area was set to 1 mm square. The PID signal was visualized using NanoZoomer at an excitation wavelength of 580 nm.

### Selection of core images for analysis

The slide image of WSI was separated for each core. The slide quality was checked by experienced pathologists to remove necrosis, noninvasive cancer tissue, stripped tissue, and normal epithelium that may confound the analysis.

### Quantification of the PID signal for each core

PID fluorescence intensity (PID value) was calculated for each core. The PID value is a measure to convert classic IHC into a more quantitative range. To distinguish between invasive and non-invasive cancer areas, an experienced pathologist masked the invasive cancer area from the hematoxylin image and measured the fluorescence intensity of the PID corresponding to the invasive cancer area by combining it with the PID image. The fluorescence image was divided into 12-μm square sections at the invasive cancer region, and each section was quantified by integrating the fluorescence intensity. Because the HER2 IHC score was defined by membrane staining observed in >10% of tumor cells in a clinical guideline for HER2 testing, the PID value was calculated as the mean of the top 10% of the integrated values of the sectioned invasive cancer areas [4].

### Statistical analysis

F-tests were performed to compare areas, and equal variance was defined as p ≥0.05. Comparisons between the groups were performed using parametric Student’s t-tests (p ≥ 0.05 in the F-test) or Welch’s t-tests (p < 0.05 in the F-test). A p <0.05 was considered significant for both t-tests.

### Ethics statement

We obtained permission from Institutional Review Board of National Defense Medical College as clinical utility research of protein quantification method using fluorescent nanoparticles. The tissue microarray used in this study is already an ethically approved product from US Biomax (TissueArray.Com LLC) in the United States. Breast tumor tissues were obtained from US Biomax of tissue microarray vendor. All tissue is collected under the highest ethical standards with the donor being informed completely and with their consent. US biomax follows standard medical care and protect the donors’ privacy. All human tissues are collected under HIPPA-approved protocols (https://www.tissuearray.com/FAQs#q10).

## Results

### IHC scoring of TMA by 3, 3’-diaminobenzidine (DAB) IHC staining

**Fig 1:**
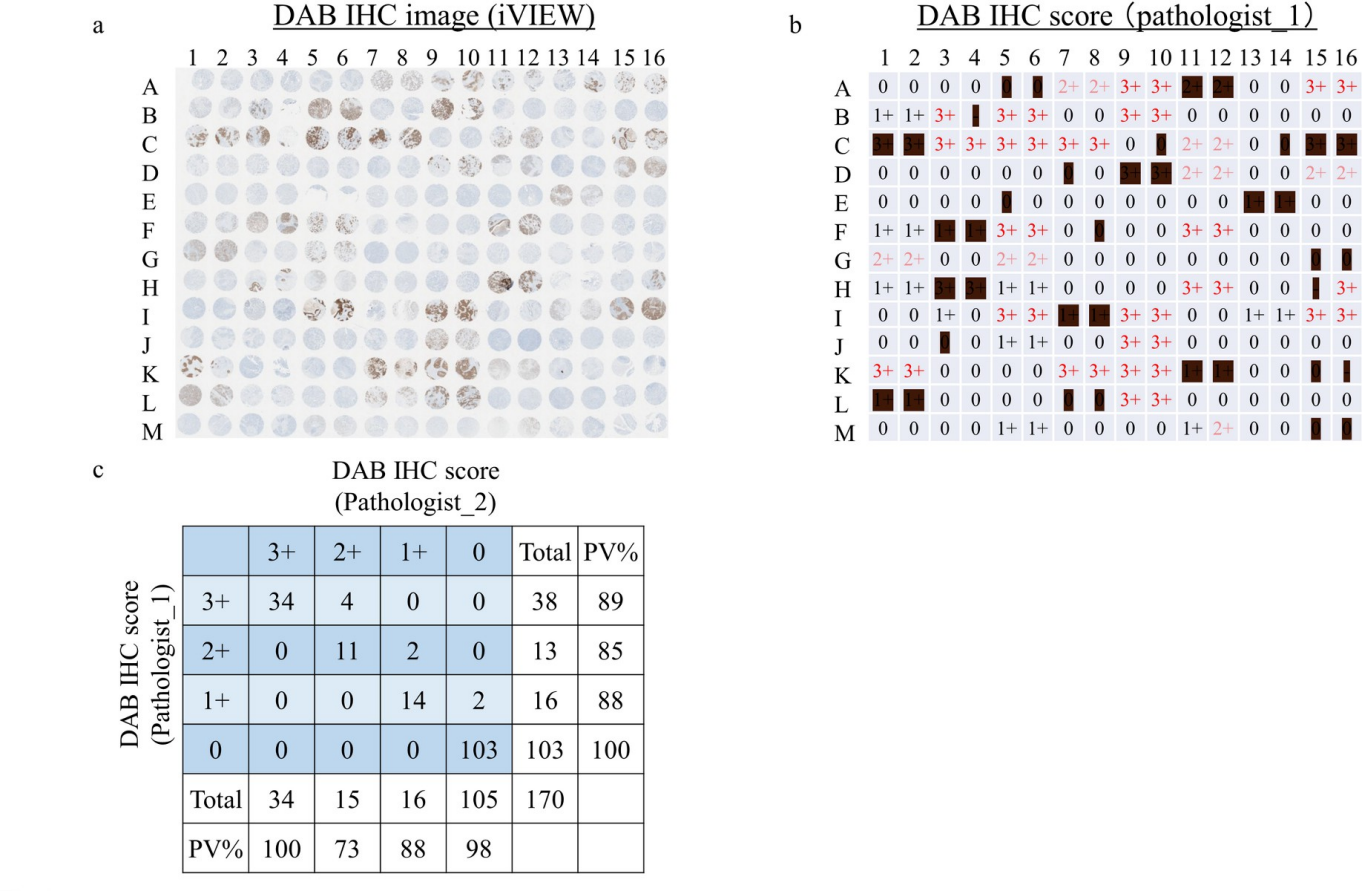
TMA map and HER2 IHC scoring by DAB staining. (a) 3,3-diaminobenzidine (DAB) immunohistochemistry (IHC) staining of HER2 in the tissue microarray cohort. (b) IHC score by experienced pathologists. The excluded core has been partially blacked out. (c) Comparison of IHC scores between experienced pathologists. PV%: Percentage of positive values.

To evaluate PID IHC staining, IHC scoring of TMA was first performed by DAB IHC staining. The breast tumor TMA included 104 cases of malignant tumors, with duplicate cores per case. DAB IHC staining of TMA was performed using the Ventana iView DAB detection kit (Fig 1A), and invasive ductal carcinoma was scored by experienced pathologists (Fig 1B). Of the 208 cores, 38 (18%) were excluded because they were not suitable for evaluation: ductal carcinoma in situ (23 cores; A5, A6, A11, A12, C1, C2, C10, C14, C15, C16, D7, D9, D10, F8, G15, G16, H3, H4, I7, I8, J3, M15 and M16), DAB judgment not possible by insufficient fixation (8 cores; F3, F4, E13, E14, K11, K12, L1 and L2), cytoplasmic staining (2 cores; L7 and L8), no cancer (3 cores; H15, K15 and K16), and core peeling (2 cores; B4 and E5). The HER2 expression in the breast tumor TMA ranges from undetectable with current HER2 IHC (IHC_0) to strongly positive (IHC_3+). The IHC score of pathologist_1 was nearly the same as that of pathologist_2 (Fig 1C). This indicates high accuracy of pathological diagnosis.

### Validation of TMA as the HER2 assay by PID

**Fig 2:**
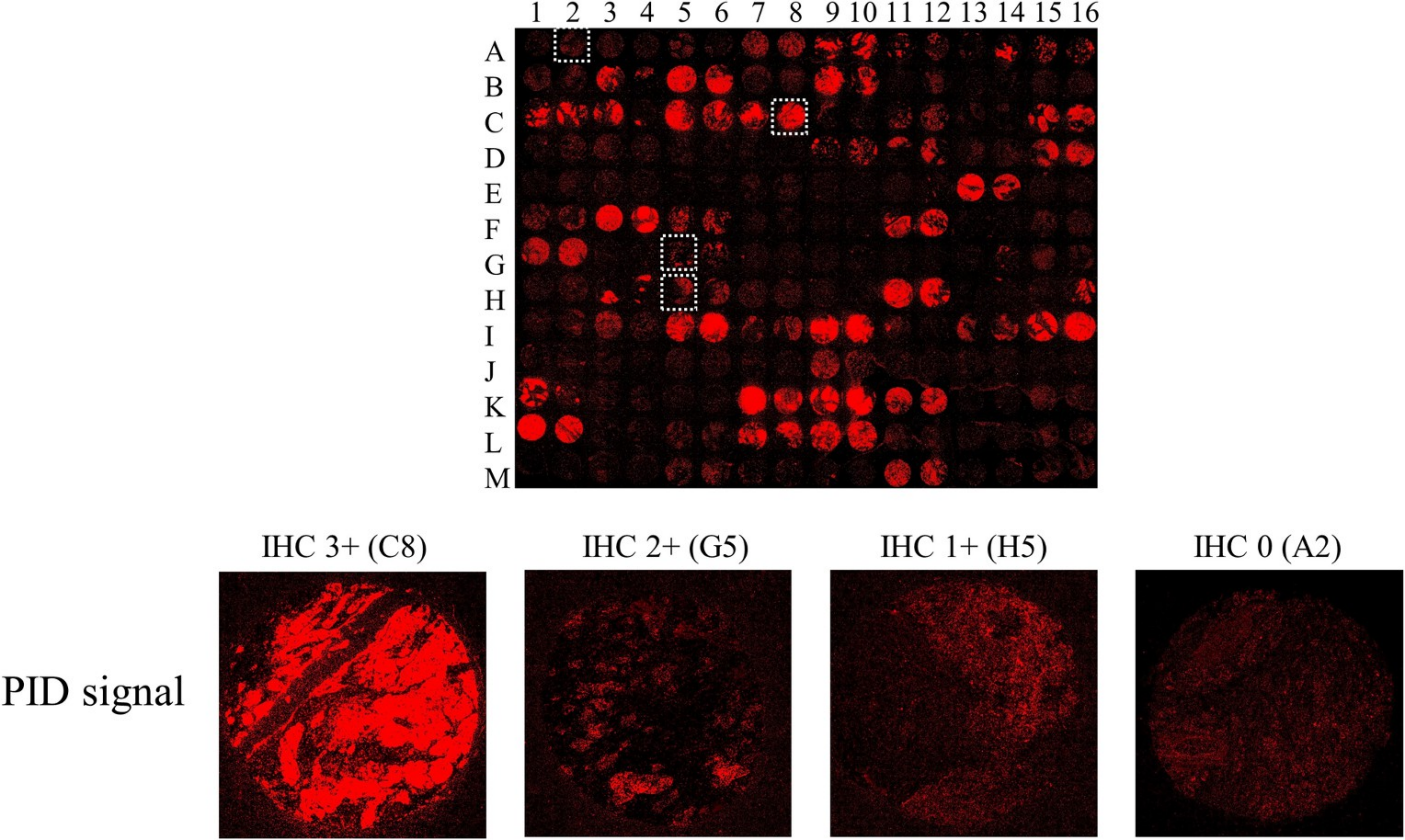
Breast tumor tissue microarray by PID staining. Representative image of TMA-stained HER2 IHC assay by phosphor-integrated dots (PID), including four spots of the negative spot (IHC 0, A2), low expresser (IHC 1+, H5), medium expresser (IHC 2+, G5), and high expresser (IHC 3+, C8). The staining images are 3× digital image magnifications on a Nanozoomer S60.

All Food and Drug Administration-approved HER2 IHC diagnostic assays are semiquantitative assessments with DAB IHC staining. HER2 IHC assays for direct HER2 quantification are currently under investigation. In this study, we used BC tissues to validate a quantitative test with fluorescent nanoparticles PID. Previously, we reported that the PID particle number estimated by IHC PIDs of BC tissues obtained from biopsy before chemotherapy can provide a score for predicting the therapeutic effect of the HER2-targeted drug trastuzumab [22]. In the present study, HER2-overexpressing (3+) and low-expressing (1+/0) samples can be observed under the same staining conditions even with TMA containing many tissues with different HER2 expression levels (Fig 2).

### Comparison between DAB IHC staining and PID IHC staining in tissues with different HER2 IHC scores (3+, 2+, 1+, and 0)

**Fig 3:**
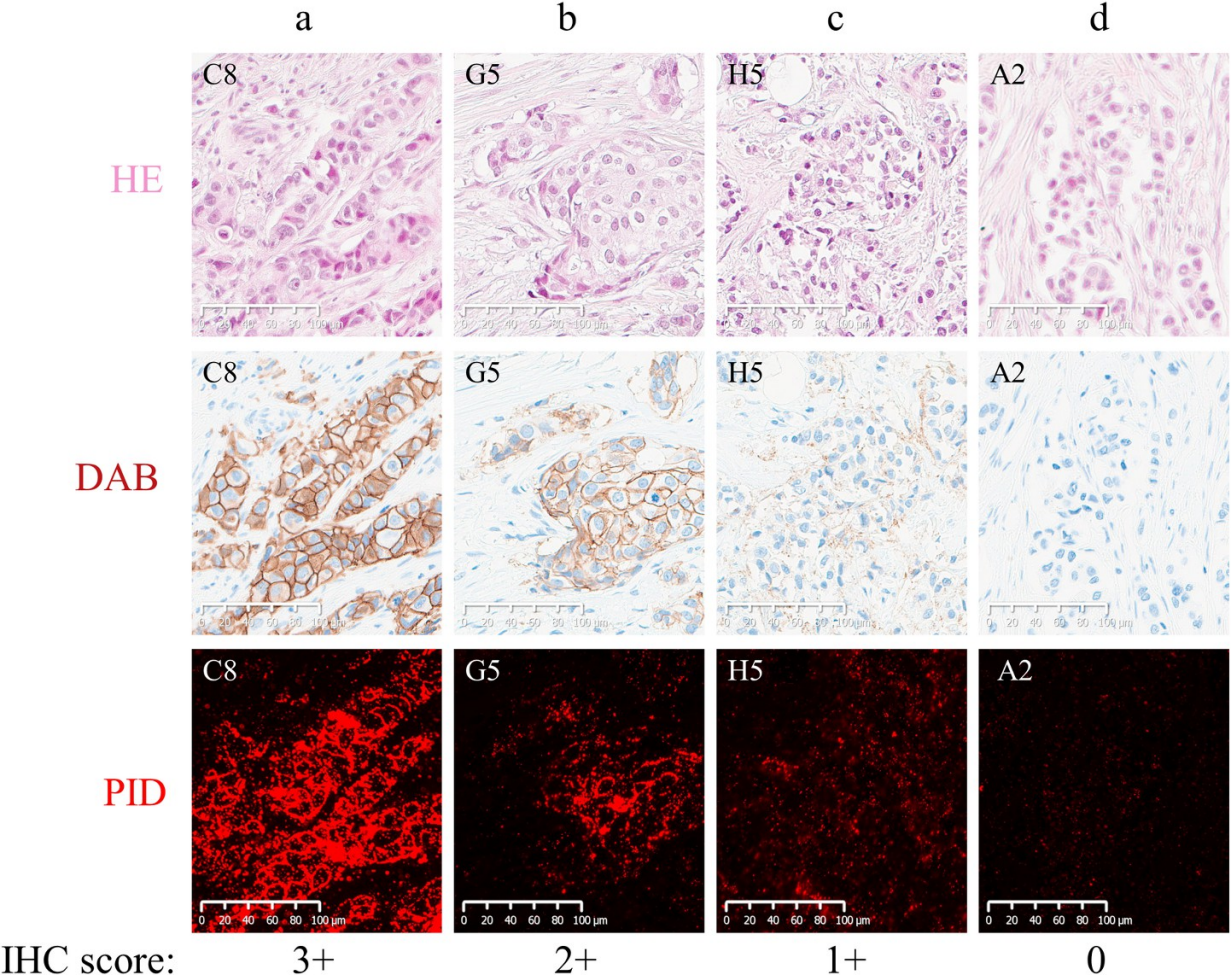
HER2-expressing breast cancer specimen stained with hematoxylin-eosin (HE) to the upper, HER2 IHC by DAB to the middle, and HER2 IHC by PID to the lower. Tissues were classified as follows: (a) 3+, (b) 2+, (c) 1+, (d) 0. The staining images are 20× digital image magnifications on a Nanozoomer S60.

Since DAB IHC staining of HER2 1+/0 shows a weak signal, making an objective and reproducible judgment is not easy. However, with PID IHC staining, a clear positive signal can be detected even in cores classified as HER2 1+ by DAB IHC staining. Moreover, PID IHC staining was also observed with cores that were classified as HER2 0 expression with DAB IHC staining (Fig 3). HER2 IHC_0 had more bright spots than the HER2-negative control (S1A Fig). Furthermore, PID IHC staining showed similar staining to DAB in the BC cell line and the HER2-low-expressing cell line (S2 Fig). Previous reports have also demonstrated the specificity of PID immunostaining using HER2-negative control cells other than breast cancer cell lines [27].

### Approaches to classify and quantify IHC staining

**Fig 4:**
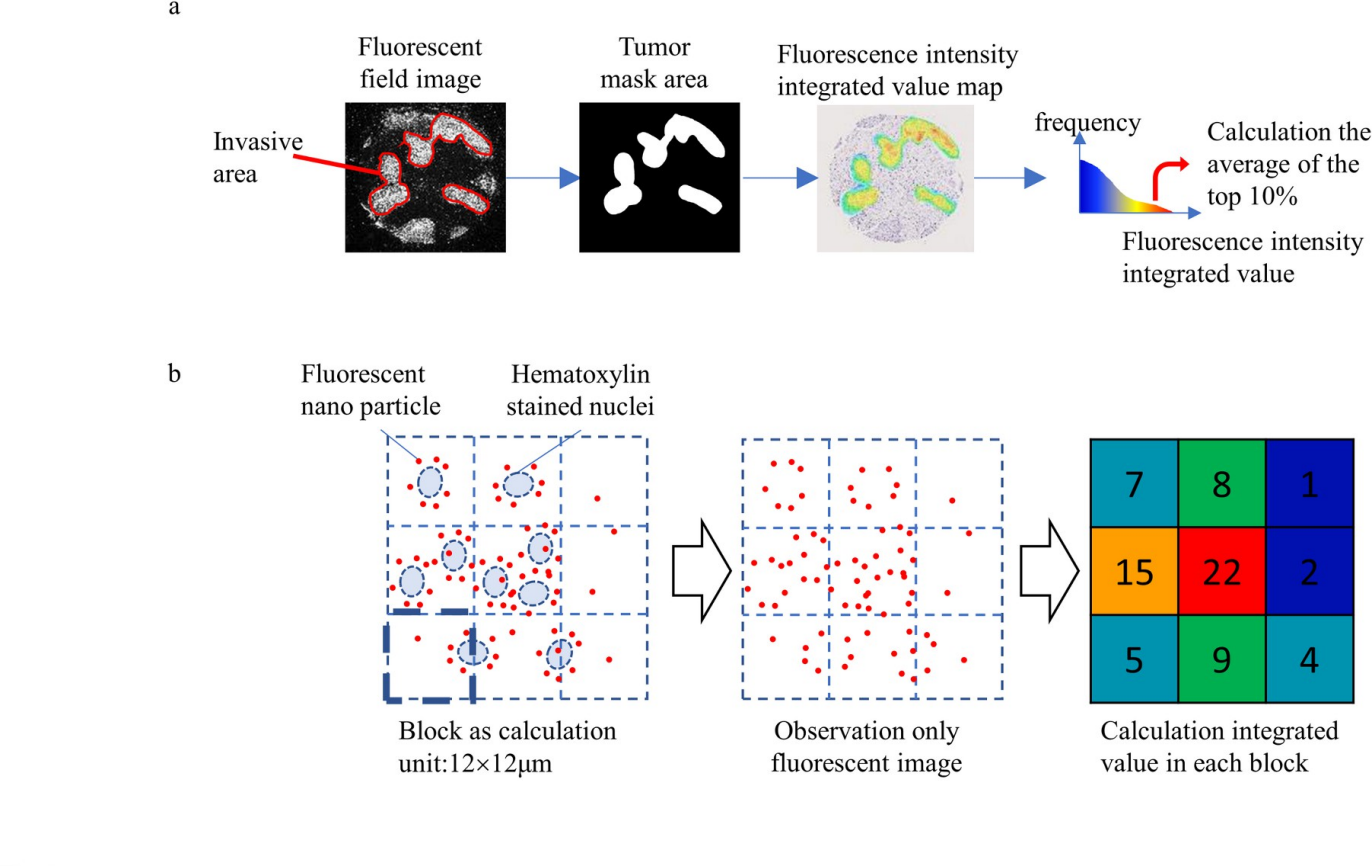
Calculation method of the integrated fluorescence intensity of HER2 expression in invasive ductal carcinoma. (a) Calculation method of PID fluorescence intensity in the invasive area. The PID value of the invasive cancer area was calculated by superimposing the invasive cancer area extracted from the hematoxylin image and PID image. (b) To measure PID fluorescence intensity, the fluorescence image of the invasive cancer region was divided into 12-μm square sections, and each section was quantified by integrating the fluorescence intensity.

Previous studies have performed PID analysis using randomly selected fields from invasive tumors [22, 28]. Because this is a partial assessment of cores, the PID value may fluctuate depending on the selection part. For a comprehensive assessment of the whole core, the PID-stained image was converted into a high-precision WSI by a virtual slide scanner, and total fluorescence intensity was extracted from the masked invasive cancer area. The IHC score was determined by a pathologist’s semiquantitative classification according to the ASCO/CAP guideline of 10% of membrane staining, whereas the PID value was quantitatively calculated from the mean fluorescence intensity of the top 10% in the invasive area to emphasize objectivity (Fig 4A). The PID calculation was based on an idea from Aperio Technology, which develops open-source software for IHC image analysis [29]. Aperio’s membrane algorithm calculates the average intensity and the completeness of membrane staining in IHC-stained slides, using the level of completeness for the top 10% as metric [30]. The integrated fluorescence intensity of PID was calculated in the extracted invasive cancer area (Fig 4B).

### Concordance in HER2 score between DAB IHC and PID IHC

**Fig 5:**
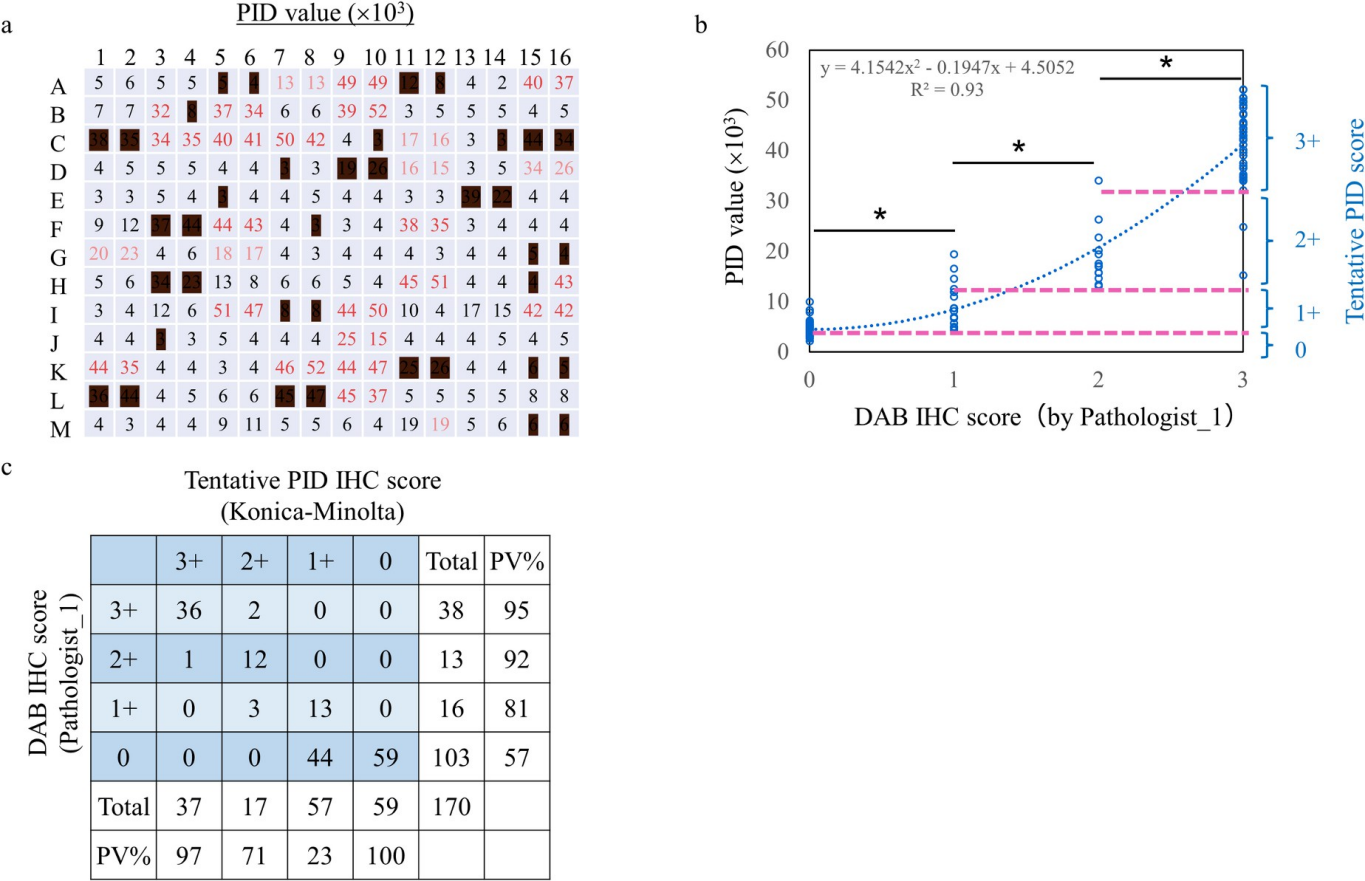
HER2 concordance between PID and DAB IHC. (a) PID value map (×10^3^) on the invasive cancer area. (b) Comparison of PID and DAB IHC (R^2^ = 0.93). The pink line shows the cut point (lowest values of 1+ (4.33 × 10^3^), 2+ (13.12 × 10^3^), or 3+ (32.17 × 10^3^)). The asterisks represent statistical significance based on two-tailed paired Student’s t-test between denoted samples (*P < 0.001). (c) Raw categorical data displaying the graph in (b). PV%: Percentage of positive values.

PID fluorescence intensity (PID value) was calculated for each core with our quantitative digital image analysis (Fig 5A). Since the PID value calculated the scores based on the fluorescence intensity, a linear score can be obtained, which is continuous rather than a discontinuous score such as 3+/2+/1+/0. The PID value showed highly concordant results when compared with the IHC score derived by pathologists (R^2^ = 0.93) (Fig 5B). Furthermore, the PID value and pathologist-derived IHC scores were compared to assess concordance between the two systems (Fig 5C). The tentative PID IHC score was determined by setting the lowest value of each DAB IHC score as the cut point. Both DAB IHC and PID IHC showed high concordance at high HER2 expression (3+ and 2+), but low prediction accuracy at low HER2 expression (1+), because DAB IHC score_0 was subdivided into PID IHC score_0 (59 cores) and PID IHC score_1+ (44 cores) by tentative PID scores. PID IHC score_1+ included cores with PID values higher than the negative control mean of 3.1 × 10^3^ (S1B and S1C Fig). Consecutive formalin-fixed, paraffin-embedded sections were tested in triplicates with PID and confirmed to be reproducible using the same HER2 antibody and antigen retrieval (S3 Fig). Reproducibility is an important factor to increase the reliability of this method. Although the stained images may differ between successive sections even in the same specimen, when comparing the IHC score derived by the pathologist and PID value, the results showed very good agreement (R^2^ = 0.93, 0.90, 0.92), indicating high detection accuracy.

### Separation ability of DAB IHC and PID IHC for low HER2 expression tumors

**Fig 6:**
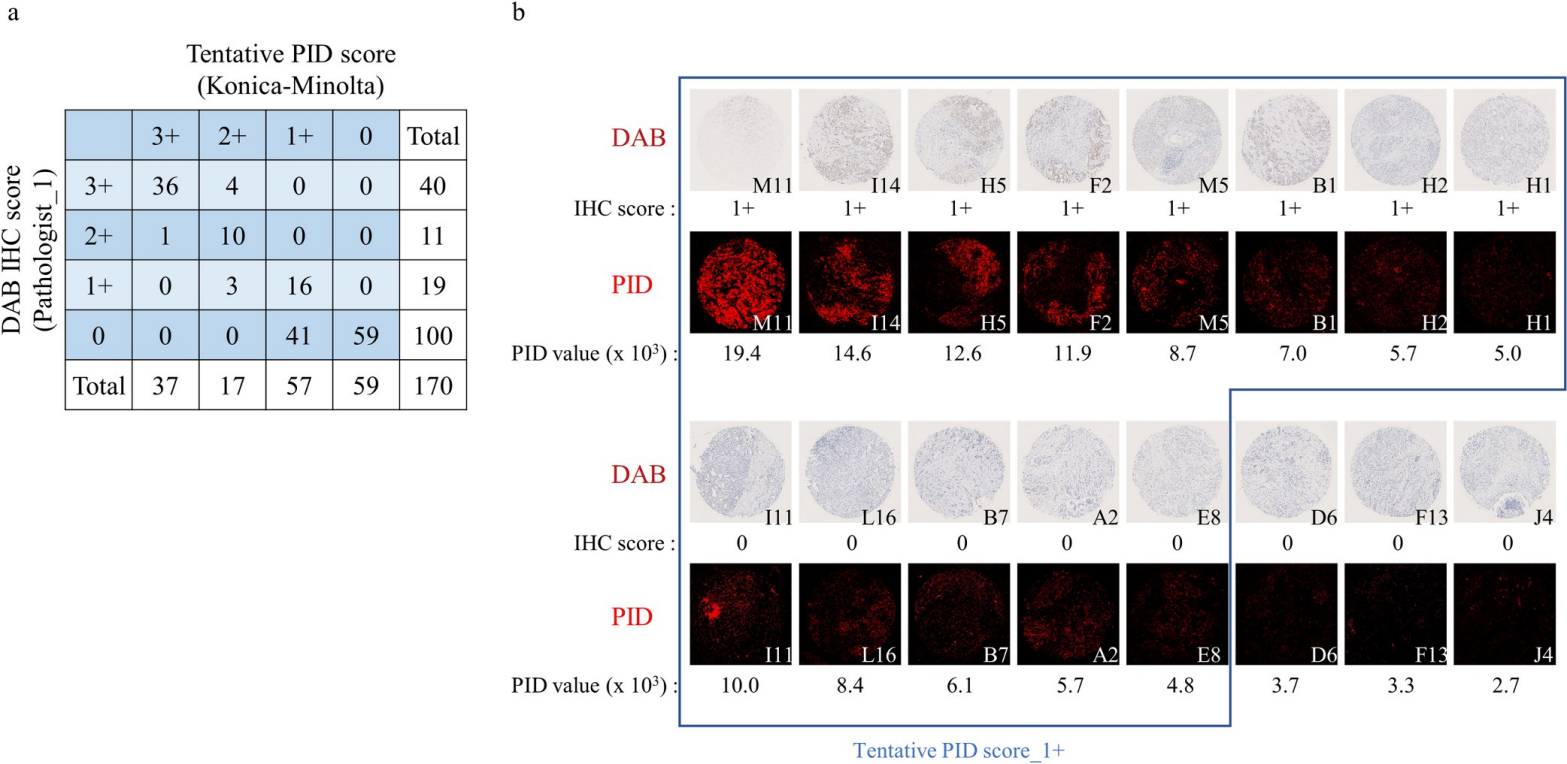
Separation ability of the low HER2 expression region. (a) HER2 concordance between DAB and PID re-evaluated based on low HER2 expression. (b) A PID-stained image of tissues judged as having low HER2 expression by DAB staining. The PID value is an integral of the fluorescence intensity of PID in the invasive cancer area. Clear positive signals were detected in all cores, classified as HER2 1+, which is difficult to determine by DAB staining. The staining images are 3× digital image magnifications on a NanoZoomer S60.

To assess the ability to separate low HER2 expression, the TMA was re-evaluated by an experienced pathologist according to the criteria for low expression. The re-evaluated classifications differed slightly from the originally evaluated classifications (Figs 5C and 6A). However, as a limitation of this assessment, conventional HER2 staining methods were used rather than approved staining methods for low HER2 expression. Since the DAB IHC scoring was performed by visual semiquantitative measurement, the score was likely to differ depending on the experience of the pathologist, particularly in the sample with low HER2 expression. DAB IHC score_0 included a core with a value higher than the lowest PID value among the tentative PID scores of 1+ (Fig 6B). PID IHC staining is a quantitative and objective evaluation based on fluorescence intensity and demonstrates the ability to numerically evaluate regions with low-expression levels that are difficult to separate by DAB IHC staining (Fig 6). The 41 cores in DAB IHC score_0 may be classified into the low HER2 category.

### Role of PID IHC within DAB IHC

**Fig 7:**
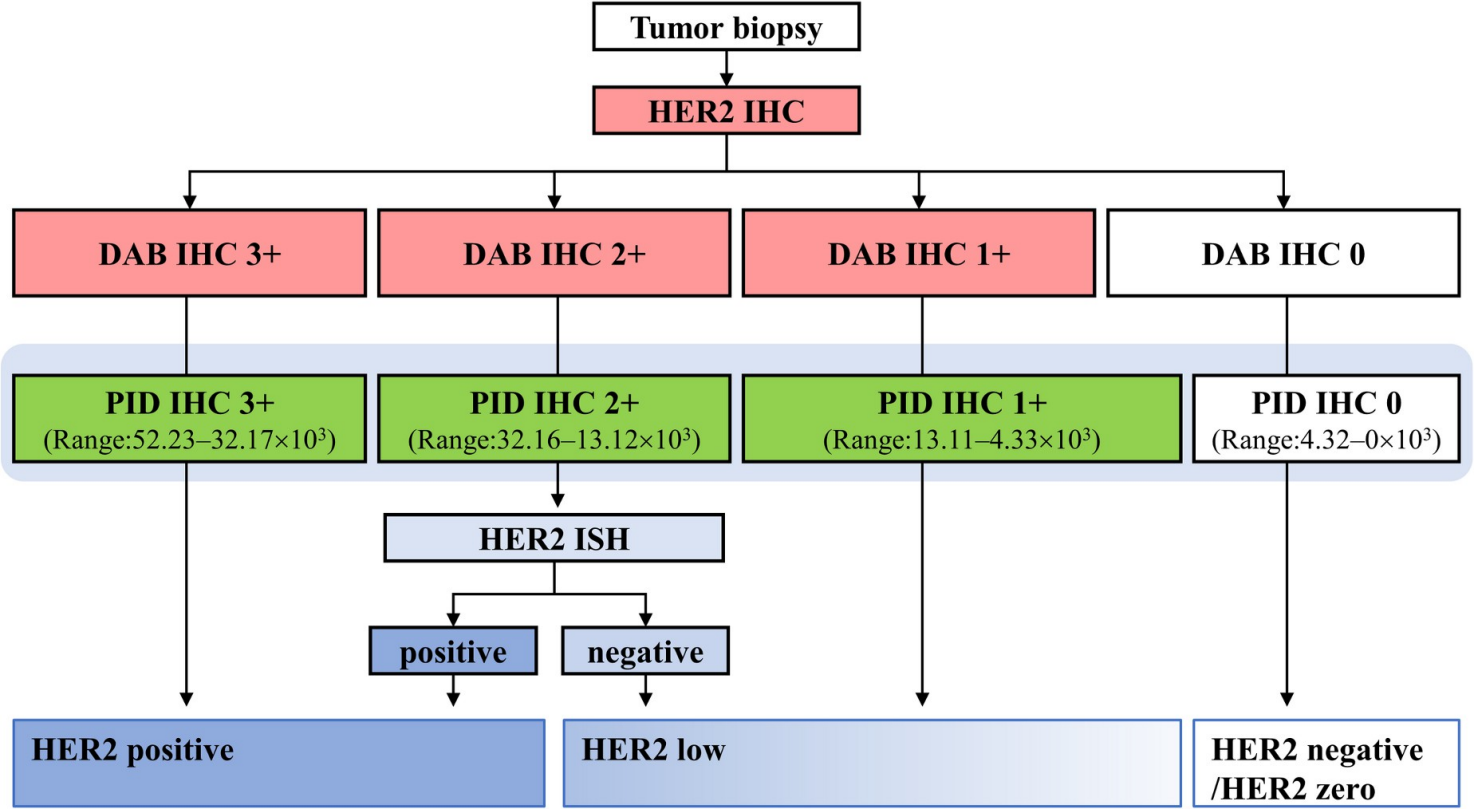
Schematic representation in the case of applying tentative PID IHC score to DAB IHC diagnosis. This shows the relationship between current HER2 DAB IHC diagnosis and PID IHC score in breast cancer. PID IHC helps classify HER2 positive, low HER2 and HER2 negative. ISH: in situ hybridization.

Fig 7 shows that the DAB IHC scores can be converted into tentative PID IHC scores and classified into four ranges. A case with a DAB IHC score of 3+ had a range of 52.23–32.17 × 10^3^ with PID IHC, a case with a DAB IHC score of 2+ had a range of 32.16–13.12 × 10^3^ with PID IHC, a case with a DAB IHC score of 1+ had a range of 13.11–4.33 × 10^3^ with PID IHC, and cases with a DAB IHC score of 0 had a range of 4.32–0 × 10^3^ with PID IHC. This shows that PID IHC increases the reliability of HER2 positive and helps distinguish between low HER2 and HER2 negative.

## Discussion

This study combined PID and WSI to assess and quantitatively classify HER2 expression from the whole core. Our findings present a high-sensitive assay for measuring HER2 expression.

This study has five major findings. First, WSI can help decrease the subjectivity from viewing only a small portion of a sample on a glass slide. In previous studies, the PID/cell (PID score/cells) and PID/unit area (100 μm^2^) (PID score/ROI 100 μm^2^) were calculated in five randomly selected fields from an invasive tumor [22, 28]. However, this method provides only partial information, and the results may contain selection bias in the area of measurement. The WSI provides comprehensive information, and the results can be objective. In addition, although the area of invasive cancer contained in each core is different, it is possible to diagnose even cores with small areas of invasive cancer by extracting the area using WSI and calculating the PID score.

Second, stratification can be performed using antibodies utilized in diagnostic tests for HER2 therapy. The VENTANA PATHWAY anti-HER2/neu (4B5) rabbit monoclonal primary antibody has been approved by the FDA as the first companion diagnostic test to identify patients with low HER2 BC for ADCs [31]. In the DESTINY-Breast04 trial, the VENTANA HER2/neu (4B5) assay system was used to identify patients with low HER2 status [32]. However, this method is a high HER2 expression detection method and was not developed to detect low HER2-expressing tumors, so it suffers from poor stratification for low HER2 expression [33]. The significance of this study is to combine PID with approved antibodies to develop a new assay with a wide dynamic range.

Third, HER2 expression can be evaluated numerically. In a study using the current standard HER2 IHC assay, the scoring accuracy for HER2 IHC in the low range (0 and 1+) was poor because a low HER2 expression was not important in HER2 IHC assays in non-ADC treatments [33]. The inaccurate diagnosis may lead to the misallocation of many patients to treatment with ADCs. By using fluorescent nanoparticles and quantitative digital image analysis, 3+, 2+, 1+ and 0 may be quantitatively stratified with good reproducibility (S3 Fig). In recent years, it has been reported that there is a cohort defined as HER2 “ultra-low” within HER2_zero [34]. We suggest that approximately half (41%) of the cores judged as HER2 IHC_0 by pathologists were stratified into the HER2 “ultra-low” category (Fig 6A). The PID is a novel method to quantify fluorescent signals that enables objective patient stratification in patient samples in this low-expression region, which is difficult to visually determine. PID IHC suggests the possibility of being used as an additional diagnostic parameter for low HER2 by incorporating it into the diagnosis of DAB IHC.

Fourth, HER2 expression is observed in fluorescent spots without enzymatic amplification. Muotafi et al. employed an enzymatic amplification method using TSA; however, it has the advantage of increasing signal strength, but carries the risk of excessive tyramide deposition [14, 35]. PIDs react with antibodies via a biotin-streptavidin reaction; thus, no special conditions are required. The high sensitivity over a wide dynamic range of PIDs is thought to be useful for the accurate assessment of low-level biomolecular changes. However, the need for a fluorescence imaging system may limit the versatility of this method.

Finally, the cutoff value is important for diagnosis. The optimal cutoff value for diagnosing HER2 positivity should be linked to the treatment outcomes of ADCs. Standard HER2 IHC assays might influence the testing sensitivity and reproducibility in distinguishing between HER2-low (IHC score 1+) and HER2-zero (IHC score 0) [36]. This study uses the lowest PID value among the DAB IHC scores as the cutoff value for PID IHC score classification. HER2 “ultra-low” (IHC score 0) could be distinguished from negative controls. Therefore, the HER2 “ultra-low” case included in HER2 score 0 can be classified (Fig 6). When used in clinical trials, the cutoff value must be determined by correlating the drug-effective patients with the PID score.

This study has some limitations. The HER2 tests were conducted according to a conventional protocol, which is different from the globally approved protocol for low HER2 expression. HER2 DAB IHC staining was performed with an automatic staining machine; however, HER2 PID IHC staining was performed manually. A fluorescence imaging system is required to calculate PID values.

In conclusion, we developed a quantification method that reflects the entire invasive area rather than using only five fields of view for digital image analysis using PID and WSI. This method can be used as a supplementary diagnostic tool to increase consistency in HER2 assessments. Future applications of PID for HER2 diagnosis need to be evaluated in clinical trials using FDA-approved companion diagnostic tests for low HER2 expression.

## Supporting information

S1 Fig Staining image and concordance table of the negative control. (a) Negative control omitting the primary antibody. The staining images are 20× digital image magnifications on a Nanozoomer S60. (b) PID value map (×10^3^) of the negative control. (c) Comparison graph of PID and DAB IHC of the negative control.
(TIF)

S2 Fig Breast cancer cell lines with different HER2 expressions. The staining images are 20× digital image magnifications on a Nanozoomer S60.
(TIF)

S3 Fig Within-run reproducibility (n = 3). (a, c, e) Comparison graph of the IHC score and PID fluorescence intensity. (b, d, f) Concordance table comparing DAB and PID. (a, b) N = 1. (c, d) N = 2. (e, f) N = 3. The asterisks represent statistical significance based on two-tailed paired Student’s t-test between the denoted samples (*P < 0.001).
(TIF)

S1 Dataset (XLSX)
