# Supplementary figures and images for: Novel approach to HER2 quantification using phosphor-integrated dots in human breast invasive cancer microarray
Source: PLoS One. 2024 May 15;19(5):e0303614. doi: 10.1371/journal.pone.0303614 (PMC11095758; doi:10.1371/journal.pone.0303614)

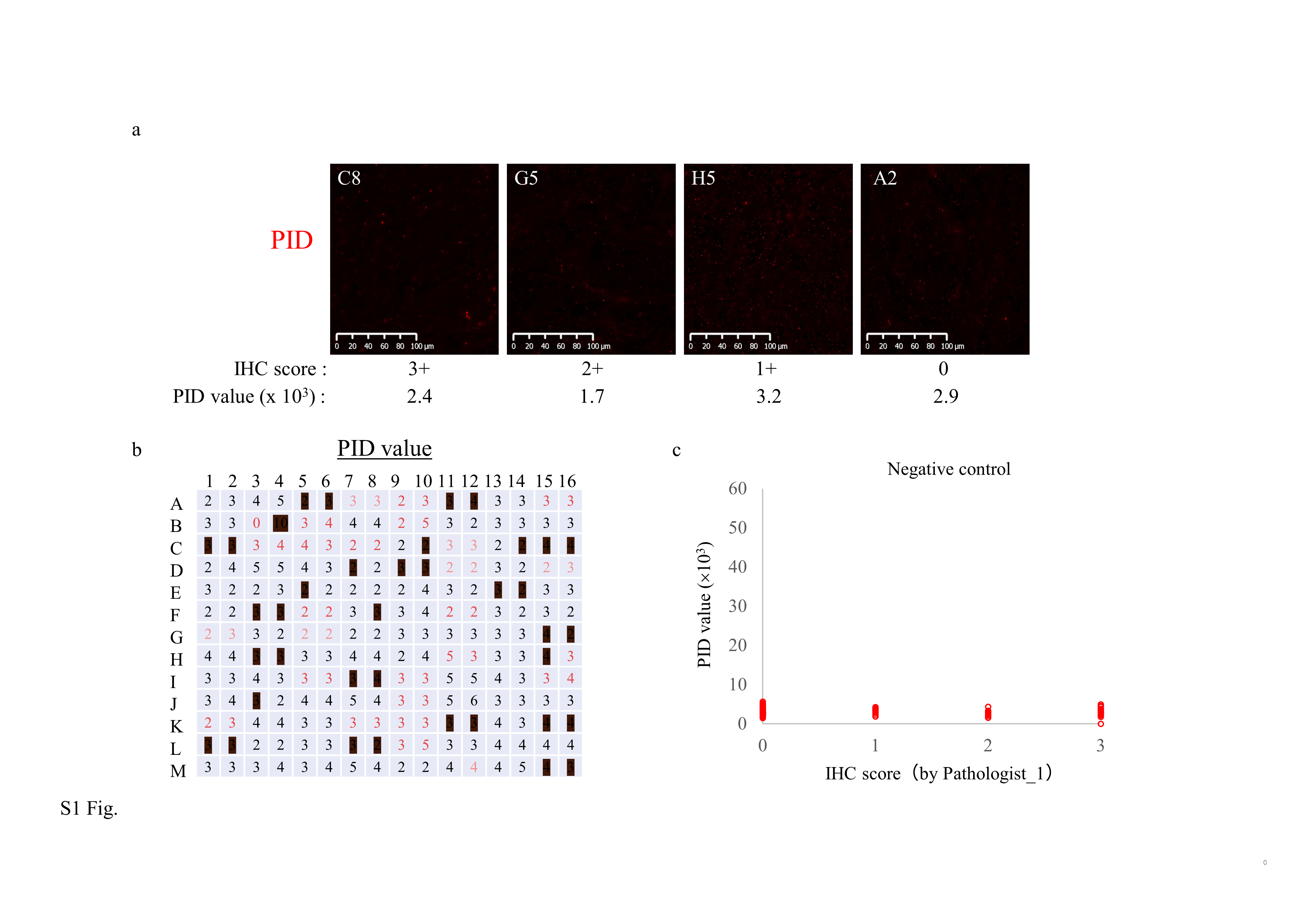

Supplement: S1 Fig — (a) Negative control omitting the primary antibody. The staining images are 20× digital image magnifications on a Nanozoomer S60. (b) PID value map (×103) of the negative control. (c) Comparison graph of PID and DAB IHC of the negative control. (TIF) [file pone.0303614.s001.tif]

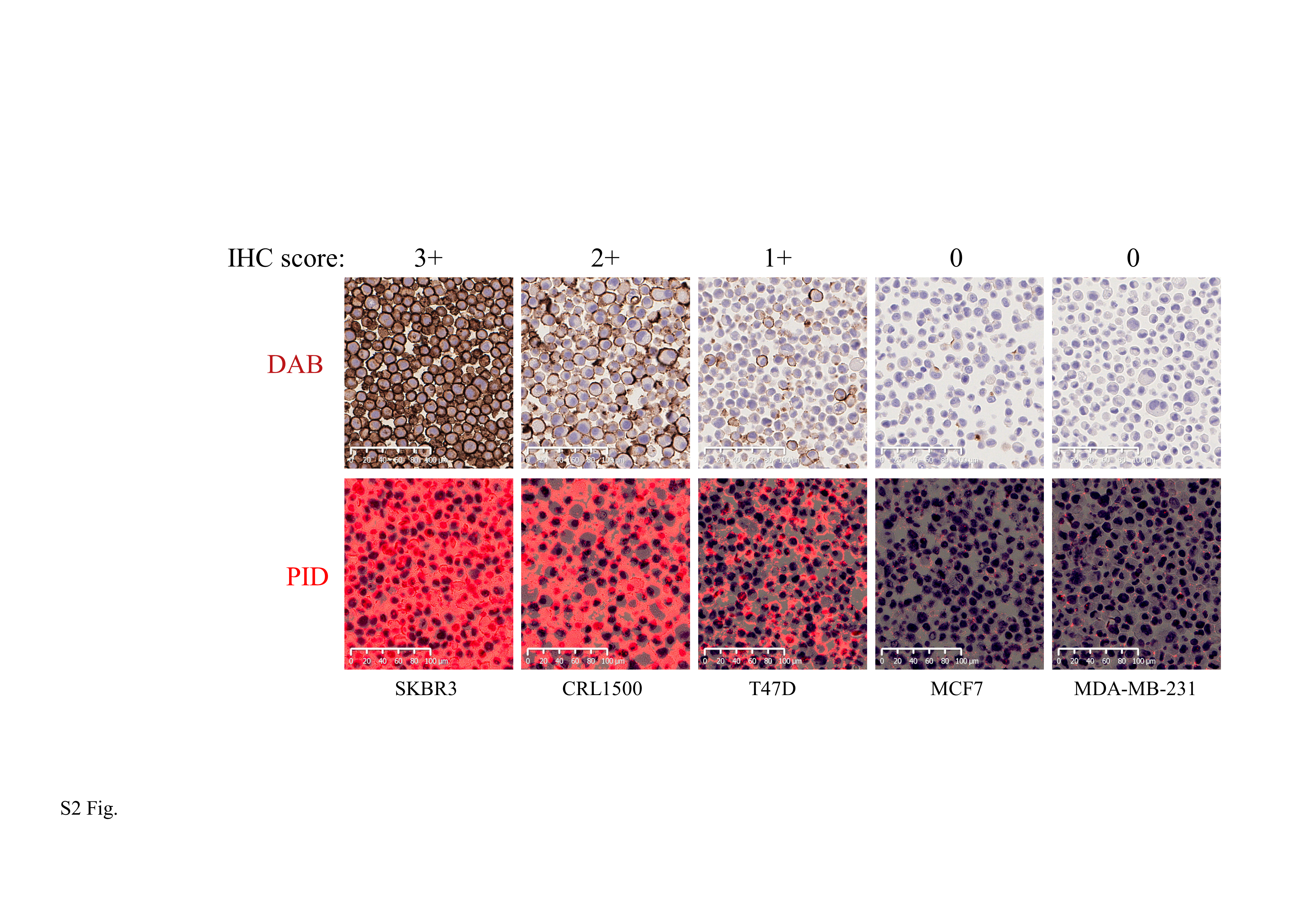

Supplement: S2 Fig — The staining images are 20× digital image magnifications on a Nanozoomer S60. (TIF) [file pone.0303614.s002.tif]

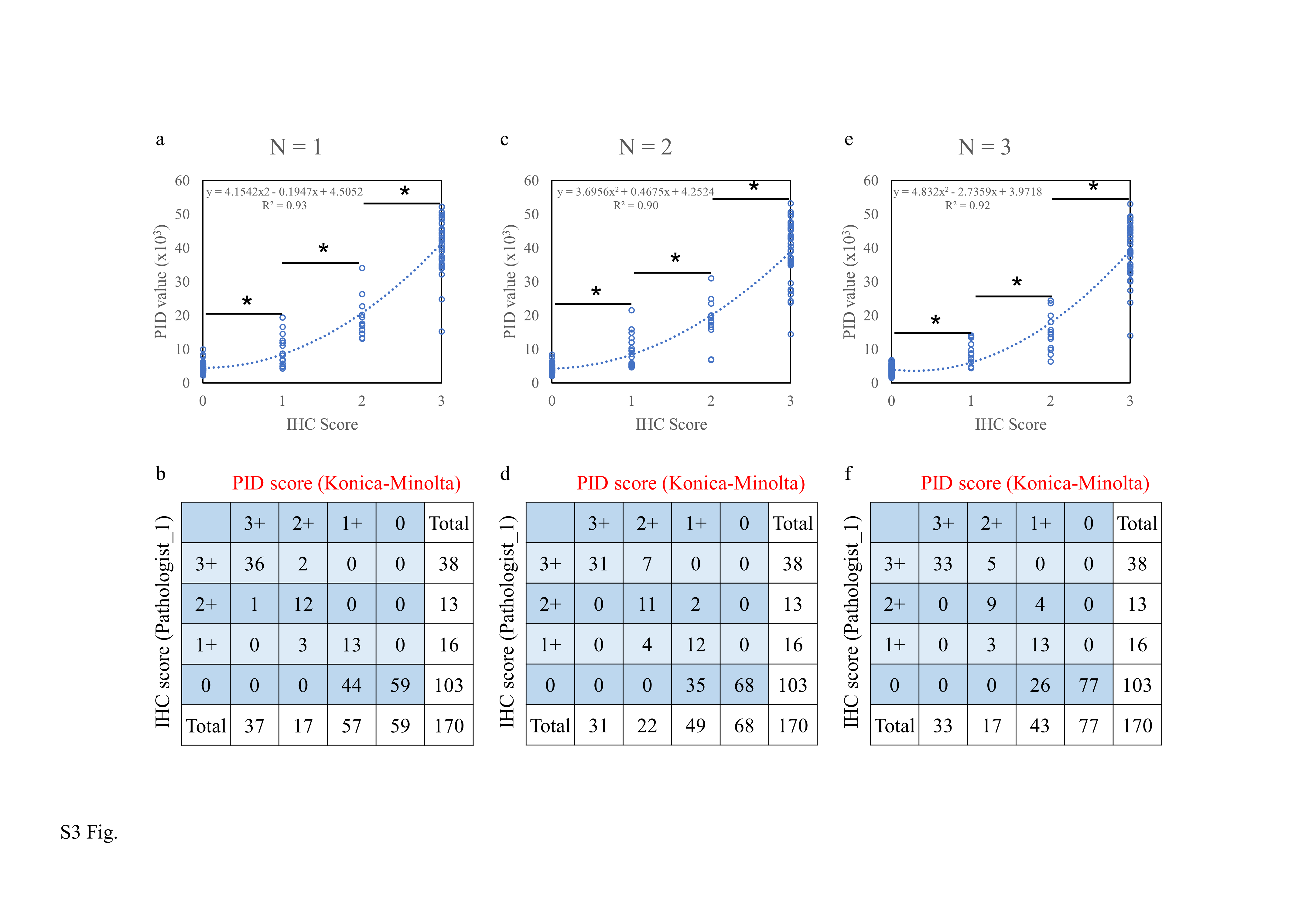

Supplement: S3 Fig — (a, c, e) Comparison graph of the IHC score and PID fluorescence intensity. (b, d, f) Concordance table comparing DAB and PID. (a, b) N = 1. (c, d) N = 2. (e, f) N = 3. The asterisks represent statistical significance based on two-tailed paired Student’s t-test between the denoted samples (*P < 0.001). (TIF) [file pone.0303614.s003.tif]
